# Integrative Gynecology and Women's Healthcare

**DOI:** 10.1155/2015/852615

**Published:** 2015-03-05

**Authors:** J. N. Lai, P. C. Chen, J. D. Wang, T. C. J. Wu, Vincent Chung

**Affiliations:** ^1^Institute of Traditional Medicine, School of Medicine, National Yang-Ming University, Taipei 11221, Taiwan; ^2^Department of Chinese Medicine, Taipei City Hospital, Yangming Branch, Taipei 11111, Taiwan; ^3^Institute of Occupational Medicine and Industrial Hygiene, National Taiwan University College of Public Health, Taipei 10055, Taiwan; ^4^Department of Public Health, College of Medicine, National Cheng Kung University, Tainan 70101, Taiwan; ^5^Ronald Reagan UCLA Medical Center, Los Angeles, CA 90095, USA; ^6^Jockey Club School of Public Health and Primary Care, Chinese University of Hong Kong, Shatin 999077, Hong Kong

Complementary treatment approaches have been increasingly demanded by women during the last several decades to relieve their gynecological disorders, reduce side effects of conventional hormonal or cancer treatment, and enhance health-related quality of life. As many women do not disclose their complementary therapies to their gynecologists or oncologists, treatment plans are often not coordinated. Thus, the risk of adverse events and interactions of complementary with conventional therapies may be increased. Sharing of information between providers of conventional and complementary treatments is important to optimize health outcomes and safety.

Integrative gynecology that combines conventional treatment with evidence-based complementary therapies and mind/body medicine has become a growing field of healthcare that strives to serve this demand. In integrative gynecology, conventional and complementary approaches are joined in a way that aims to optimize outcomes and prevent adverse events.

The papers selected for this special issue represent a good panel for addressing this challenge. Additionally, the selected topics and papers represent the rich and many-facet knowledge, which we have the pleasure of sharing with the readers. Because of limited space, we can allow only a part of gynecological studies with high quality to be published in this special issue. More research and publication space are definitely needed for integrative gynecology in the future.

The special issue contains ten papers. Two of them are related to bone loss. One paper introduces a screening system to identify Chinese herbs or formulae which will increase either HER2 or ER*α* protein expression of MCF-7 human breast cancer cell. Three papers are related to breast cancer risk of commonly used Chinese herbs; one of them considers the breast cancer risk of Chinese herbal remedies among hormone users, while another covers the subsequent endometrial cancer problems when tamoxifen users consume* Ginseng*. One paper focuses on the ovarian cancer risk of the herb-drug interaction between* fermented wheat germ extract* and cisplatin or docetaxel. One paper provides precious observational result of the effects of passive hydrotherapy among women in their ≥34th week of gestation. One paper tries to disclose the possible mechanism of Chinese herb for treating endometriosis. Finally, one paper introduces a practical method for studying the effectiveness and safety of traditional Chinese remedies for women with dysmenorrhea.

In a paper titled “*Bian Zheng Lun Zhi* as a Complementary and Alternative Treatment for Menstrual Cramps in Women with Dysmenorrhea: A Prospective Clinical Observation,” P.-Y. Lin et al. explore questions related to the strengths, weaknesses, challenges, and accomplishments of TCM treatments based on* Bian Zheng Lun Zhi* theory, along with the results of active surveillance proactively in women with primary dysmenorrhea or endometriosis.

The following three articles focus on the observational evaluation of integrative and/or complementary therapies in human populations. In a paper titled “Concurrent Use in Taiwan of Chinese Herbal Medicine Therapies among Hormone Users Aged 55 Years to 79 Years and Its Association with Breast Cancer Risk: A Population-Based Study,” Y.-T. Tsai et al. evaluate the breast cancer risk of consuming Chinese herbs, especially the extent to which the hormone users used Chinese herbs concurrently. In another paper titled “The Prescription Pattern of Chinese Herbal Products Containing* Ginseng* among Tamoxifen-Treated Female Breast Cancer Survivors in Taiwan: A Population-Based Study,” W.-L. Hsu et al. investigate the inhibitory relationship between* Ginseng* consumption and subsequent endometrial cancer among tamoxifen users. In a third article titled “Effects of Passive Hydrotherapy WATSU (WaterShiatsu) in the Third Trimester of Pregnancy: Results of a Controlled Pilot Study,” A. M. Schitter et al. carry out a controlled pilot study to assess the potential therapeutic effects of passive hydrotherapy on self-reported stress, pregnancy-related pain, mood and quality of life, amount of amniotic fluid, blood flow characteristics, spontaneous course of breech presentations, and prospects of external cephalic versions in women in their ≥34th week of gestation.

In a paper titled “Preventive Effects of Collagen Peptide from Deer Sinew on Bone Loss in Ovariectomized Rats,” H. Zhang et al. test the preventive effect of deer sinew on ovariectomy-induced bone loss. In a paper titled “Prolonged Diuretic Activity and Calcium-Sparing Effect of* Tropaeolum majus*: Evidence in the Prevention of Osteoporosis,” L. N. Barboza et al. are considering the diuretic effects of prolonged administration of* Tropaeolum majus* in ovariectomized rats and the interrelationship between calcium excretion and bone turnover.

In a paper titled “Propolis is an Efficient Fungicide and Inhibitor of Biofilm Production by Vaginal* Candida albicans*,” I. R. G. Capoci et al. test the minimum inhibitory concentration and the minimum fungicidal concentration and assess the cell viability, protein, and carbohydrate characteristics of the biofilm of clinical isolates from vulvovaginal candidiasis to* Propolis*.

